# Diagnostic value of α1-MG and URBP in early diabetic renal impairment

**DOI:** 10.3389/fphys.2023.1173982

**Published:** 2023-10-19

**Authors:** Yukun Zhou, Yiding Zhang, Jiaojiao Chen, Ting Wang, Huangmin Li, Feng Wu, Jin Shang, Zhanzheng Zhao

**Affiliations:** ^1^ Department of Nephrology, The First Affiliated Hospital of Zhengzhou University, Zhengzhou, China; ^2^ School of Medicine, Zhengzhou University, Zhengzhou, Henan, China; ^3^ Laboratory of Nephrology, The First Affiliated Hospital of Zhengzhou University, Zhengzhou, Henan, China; ^4^ Laboratory Animal Platform of Academy of Medical Sciences, Zhengzhou University, Zhengzhou, Henan, China

**Keywords:** diabetic kidney disease, tubular injury markers, type 2 diabetes mellitus, diagnostic model, renal prognosis

## Abstract

**Aims/Introduction:** Diabetic kidney disease (DKD) is defined as diabetes with impaired renal function, elevated urinary albumin excretion, or both. DKD is one of the most common microvascular complications of diabetes and plays an important role in the cause of end-stage renal disease (ESRD). About 5% of people with type 2 diabetes (T2DM) already have kidney damage at the time they are diagnosed, but other triggers of renal insufficiency, such as obesity, hyperlipidemia, glomerular atherosclerosis are often present, making it difficult to define “diabetic kidney disease” or “diabetic nephropathy” precisely in epidemiology or clinical practice. Therefore, the aim of this study is to identify diabetic patients with CKD at an early stage, and evaluate the value of tubular injury markers including α1-microglobulin (α1-MG), β2-microglobulin (β2-MG), N-acetyl-beta-D-glucosaminidase (NAG) and Urinary retinol binding protein (URBP) in the development of diabetes to DKD.

**Materials and methods:** We recruited a total of 182 hospitalized patients with T2DM in the First Affiliated Hospital of Zhengzhou University from February 2018 to April 2023. We collected basic clinical characteristics and laboratory biochemical parameters of the patients. Based on their levels of urinary albumin creatinine ratio (UACR) and glomerular filtration rate (GFR), patients were divided into DM group (UACR≤30 mg/g and eGFR≥90 mL/min/1.73 m^2^, *n* = 63) and DKD group (UACR>30 mg/g or eGFR<90 mL/min/1.73 m^2^, *n* = 119) excluding other causes of chronic kidney disease. We further developed diagnostic models to improve the ability to predict the risk of developing DKD by screening potential risk factors using univariate and multivariate logistic regression analysis. Calibration plots and curve analysis were used to validate the model and clinical usefulness. Next, we screened patients with relatively normal estimated glomerular filtration rate (eGFR) (≥90 mL/min/1.73 m^2^) to investigate whether tubular injury markers could accurately predict the risk of DKD in patients with normal renal function. We defined the rate of GFR decline as a prognostic indicator of renal function in patients and collected the information of the re-hospitalized DKD patients to determine whether the relevant indicators had an impact on the renal prognosis.

**Results:** The patients with DKD had higher levels of tubular injury markers than patients with DM. URBP, α1-MG, eGFR were statistically different in both univariate and multivariate logistic regression analyses and displayed great predictive power after modeling with an area under curve of 0.987. The calibration curve showed medium agreement. Decision curve showed it would add more net benefits for clinical decision. After adjusting eGFR and serum creatinine (Scr), URBP was demonstrated to be associated with early renal function impairment.

**Conclusion:** Tubular injury markers play an important role in early diabetic renal function impairment.

## Introduction

About 30%–40% of patients with both type 1 diabetes (T1DM) and type 2 diabetes (T2DM) experience renal impairment (Alicic et al., 2017) and approximately 5% of patients with T2DM already have DKD at the time of diagnosis of diabetes. The most common cause of the occurrence and development of CKD and ESRD is T2DM nowadays (Afkarian et al., 2016). The natural progression of DKD includes glomerular hyperfiltration, progressive proteinuria, decreased GFR, and eventual progression to ESRD requiring renal replacement therapy, leading to great financial burden to the patients. Preventing the development and progression of chronic kidney disease or ESRD and reducing the number of patients undergoing dialysis could bring substantial benefits in reducing global healthcare costs (Foley and Collins, 2009).

In clinical work, the only gold standard for the diagnosis of diabetic nephropathy is still renal biopsy. However, it cannot be done in some individuals. As an invasive tool, renal biopsy can only be performed in those who do not have contraindications. On the other hand, renal biopsy is relatively expensive. As a result, it is still diagnosed based on its clinical presentation in most clinical work. According to relevant guidelines, screening and staging of early diabetic kidney disease is based on levels of albuminuria and eGFR (Levey et al., 2011). Especially, the albuminuria is the important diagnostic or prognostic biomarker of DKD currently used in clinical practice. However, there is still a significant proportion of patients with DKD who are negative for proteinuria (non-albuminuria phenotype), which makes it difficult to diagnose DKD early (Penno et al., 2011). Several biomarkers associated with tubular injury have been identified as potent predictors of renal outcome. It has been reported that circulating tumor necrosis factor receptors (TNFR) 1 and 2 was strongly associated with the prognosis of diabetic patients (Hwang et al., 2017). A growing body of literature supports that inflammatory processes and renal tubular damage play a significant role in the early course of DKD (Gilbert and Cooper, 1999; Thomas et al., 2005; Magri and Fava, 2009). Therefore, in this study we focus on markers of tubular injury and early detect renal injury.

As low molecular weight proteins, α1-microglobulin (α1-MG) and β2-microglobulin (β2-MG) has properties that are readily filtered by glomeruli and reabsorbed and catabolized by proximal tubular cells and as a marker of tubular function injury has also been studied in other types of diseases (Yu et al., 1983). In one study, α1-MG and β2-MG were found to be associated with eGFR independently of albuminuria, suggesting that they may play an important role in the development and progression of DKD (Jiang et al., 2018). In another American study, it was found that urinary B2-MG excretion was significantly correlated with the severity of tubulointerstitial damage in patients with DKD confirmed by renal biopsy, indicating that B2-MG also has a good predictive ability in the early stage of DKD (Mise et al., 2016a; Siddiqui et al., 2019). N-acetyl-beta-D-glucosaminidase (NAG) is a lysosomal enzyme, and in recent studies, NAG has shown a strong association with the early course of DKD. Previous studies have reported that in early-stage patients with DKD, compared with the appearance of albuminuria, it is more likely to be manifested by increased excretion of urinary NAG, so this biomarker may well predict the early occurrence of DKD before protein leakage (Nauta et al., 2011; Fu et al., 2012). Retinol-binding Protein (RBP), discovered by Berggard in 1961 in immunoelectrophoresis, can be filtered through the glomeruli, most of which is reabsorbed by proximal renal tubular epithelial cells, and only a small amount is excreted from the urine. Urinary retinol-binding protein is an indicator of early renal tubular injury in patients with multiple myeloma (Rezk et al., 2021). Meanwhile, URBP excretion was found to be increased in diabetic patients compared with healthy subjects and correlated with 24-hour urinary protein and serum creatinine. (Zahra et al., 2014). In addition, urinary RBP excretion was higher in patients with diabetic macrovascular and/or microvascular complications compared with patients without diabetic macrovascular or microvascular complications (Hong et al., 2000a; Hong et al., 2000b). This confirms that URBP has an important role in predicting renal complications in diabetic patients. URBP is also a predictor of dialysis risk, doubling of serum creatinine or death in DKD patients, suggesting that URBP can be used as a prognostic indicator for DKD patients (Titan et al., 2012).

Therefore, the aim of this study was to evaluate the role of tubular injury markers including α1-MG, β2-MG, NAG and URBP in the development from diabetes to diabetic kidney disease. By assessing individual risk factors, physicians and patients can take more timely measures on lifestyle management such as low-salt and low-fat diabetic diet and medical intervention.

## Materials and Methods

### Patients and ethics approval

In this retrospective analysis, we screened all diabetic patients who were hospitalized at the First Affiliated Hospital of Zhengzhou University from February 2018 to April 2023. Criteria for inclusion were as follows: 1) complete four markers of tubular injury, 2) age 18–80 years, 3) diagnosed as T2DM.The exclusion criteria were severe liver disease, malignancy, autoimmune disease such as systemic lupus erythematosus and schizophrenia. According to the level of UACR, they were divided into DM group (UACR≤30 mg/g and eGFR≥90 mL/min/1.73 m^2^, *n* = 63) and DKD group (UACR>30 mg/g or eGFR<60 mL/min/1.73 m^2^, *n* = 119). A total of 182 patients including 63 DM and 119 DKD were enrolled in this study.

The First Affiliated Hospital of Zhengzhou University Ethics Review Committee granted ethical approval for the study and the ethics review approval ID was “KY-2022-0529.” The informed consent was waived by the ethics commission. All methods were performed in accordance with the relevant guidelines and regulations.

### Data collection

We collected clinical features and laboratory tests from all patients. The basic information included age, gender, body mass index (BMI), systolic blood pressure (SBP) and diastolic blood pressure (DBP). The laboratory indices included Serum potassium, Serum sodium, Serum calcium, Urea nitrogen, Serum creatinine (Scr), uric acid (UA), total cholesterol (TC), high density lipoprotein (HDL), low density lipoprotein (LDL), albumin (ALB) levels, triglyceride (TG), eGFR, red blood cell (RBC) count, white blood cell (WBC) count, platelet (PLT) count, hemoglobin (Hb), 24-hour uric total protein (24-hour TP), urinary α1-MG, β2-MG, NAG, and RBP levels.

### Statistical analysis

Patients with DKD or DM were randomly assigned to a validation group (*n* = 35) and a training group (*n* = 147). Because the distribution of some continuous variables in this study was not normal, and the difference between the two groups was too large, we performed natural logarithm transformation of α1-MG, β2-MG, and URBP before analysis to reduce the error caused by the analysis. We used descriptive statistics of variables to describe the characteristics of each group. Continuous variables were described by mean and standard deviation if the data followed a normal distribution, or median and interquartile range if they did not. Data analysis was performed using R software (version 4.3.0) and SPSS 25.0.

There were a small number of missing values in this study. We used the Expectation-Maximization algorithm and the mean method to fill in missing values so that we minimized errors and improved statistical significance. We used univariate logistic regression to screen for potential risk factors and subsequently included these variables in multivariate logistic regression. The candidate variables with a *p* < 0.05 in the univariate analysis were enrolled to develop the multivariable model. And then we initially assessed the diagnostic efficacy of the model by drawing a receiver operating characteristic curve (ROC) and the area under the curve (AUC) was used to evaluate the diagnostic efficiency of the model. Calibration curves were used to evaluate the calibration of the DKD incidence risk nomogram. Next, we used the meaningful variables to draw nomograms for clinical application. Decision curve analysis (DCA) was used to verify the clinical utility of the nomogram (Huang et al., 2016). To balance the difference in glomerular filtration rate between DM and DKD groups, the propensity score matching (PSM) method was used to match the characteristics of the two groups of patients in a 1:1 ratio. Finally, Spearman correlation analysis was used to investigate the relationship between markers of tubular injury and renal prognosis.

## Results

### Patient characteristics

All screened T2DM patients were divided into DKD and DM groups according to the UACR levels. Among all patients, there were 121 males (66.5%) and 61 females (33.5%). The mean age was 54.2 ± 10.4 years in DM group and 51.6 ± 11.2 in DKD group (range 18–80 years). Tubular injury markers were significantly increased in the DKD group compared with the DM group (*p* < 0.001). Baseline characteristics for all patients are presented in Table 1.

**TABLE 1:** Differences in demographic and clinical characteristics between the DM and DKD groups in training group.

	DM group (N = 63)	DKD group (N = 119)	*p*-value
Age (years)	54.2 ± 10.4	51.6 ± 11.2	0.111
Female n (%)	24 (38.1)	37 (31.2)	0.342
body mass index (kg/m^2^)	25.29 ± 4.45	25.61 ± 3.27	0.277
Systolic blood pressure (mmHg)	128 (122,135)	143 (132,159)	<0.001
Diastolic blood pressure (mmHg)	80 (75.86)	92 (86,103)	0.004
Serum potassium (mmol/L)	4.25 (4.12,4,54)	4.69 (4.32, 5.26)	0.471
Serum sodium (mmol/L)	140 (138,142)	144 (142,146)	0.032
Serum calcium (mmol/L)	2.3 (2.21, 2.39)	2.19 (2.06, 2.31)	<0.001
Urea nitrogen (mmol/L)	5.4 (4.6, 6.8)	8.8 (5.8, 13.9)	<0.001
Serum creatinine (mmol/L)	62 (52, 73)	111 (76.246)	<0.001
Uric acid (μmol/L)	275 (223,331)	332 (292,399)	<0.001
Total cholesterol (mmol/L)	4.72 (3.98, 5.41)	4.41 (3.56, 5.89)	0.427
HDL cholesterol (mmol/L)	1.05 (0.84, 1.29)	0.96 (0.8, 1.17)	0.117
LDL cholesterol (mmol/L)	2.92 (2.07, 3.58)	2.56 (1.92, 3.60)	0.507
Albumin (g/L)	42.6 (39.7, 44.8)	36.7 (30.7, 40.2)	<0.001
Triglyceride (mmol/L)	1.42 (0.92, 2.42)	1.75 (1.04, 2.49)	0.233
eGFR (mL/min/1.73 m^2^)	106.487 (95.59,114.734)	60.34 (23.546, 89.072)	<0.001
RBC (10^12^/L)	3.83 ± 0.89	4.55 ± 0.63	<0.001
WBC (10^9^/L)	5.8 (5.19, 6.88)	6.33 (5.29, 6.77)	0.267
PLT (10^9^/L)	217 (179,244)	212 (177.256)	0.597
Hb (g/L)	136 (125.5,146)	115 (99,132)	<0.001
24hTP (g)	0.15 (0.12, 0.19)	3.07 (1.1, 6.2)	<0.001
NAG(U/L)	5 (3.5, 9.6)	12 (7.4, 19.44)	<0.001
URBP (μg/L)	5.52 (5.30, 5.60)	9.70 (8.75, 10.63)	<0.001
α1-MG (μg/L)	7.78 (7.28, 8.32)	10.09 (9.23, 11.12)	<0.001
β2-MG (μg/L)	5.08 (4.50, 6.45)	8.80 (7.74, 9.94)	<0.001^1^

LDL low density lipoprotein, HDL high density lipoprotein, eGFR estimated glomerular filtration rate, RBC red blood cell, WBC white blood cell, PLT platelet, Hb hemoglobin, 24hTP 24 h uric total protein, NAG N-acetyl-beta-D-glucosaminidase, URBP urinary retinol binding protein, α1-MG α1-microglobulin, β2-MG β2-microglobulin.

All patients with DKD and DM were randomly divided into a training group (*n* = 147) and a validation group (n = 35). There were roughly no statistically significant differences between these variables, illustrating the similar clinical profiles between the two groups in Table 2.

**TABLE 2:** Baseline characteristics showed in the training group and validation group.

	Training group (N = 147)	validation group (N = 35)	*p*-value
Age (years)	53.8 ± 10.6	51.3 ± 10.9	0.210
Female n (%)	49 (33.3)	12 (34.3)	0.915
body mass index (kg/m^2^)	24.77 (22.89, 27.36)	25.88 (24.22, 27.77)	0.130
Systolic blood pressure (mmHg)	136 (126,150)	138 (126,156)	0.645
Diastolic blood pressure (mmHg)	82 (76, 90)	84 (77, 90)	0.624
Serum potassium (mmol/L)	4.27 (3.98, 4.57)	4.32 (4.18, 4.79)	0.142
Serum sodium (mmol/L)	142 (138.4, 43)	141.9 (138,144)	0.818
Serum calcium (mmol/L)	2.22 (2.12, 2.32)	2.28 (2.17, 2.41)	0.047
Urea nitrogen (mmol/L)	6.8 (5.0, 11.2)	7.1 (5.3, 9.1)	0.704
Serum creatinine (mmol/L)	80 (61,157)	85 (63,131)	0.903
Uric acid (μmol/L)	322 (251,395)	313 (296,355)	0.684
Total cholesterol (mmol/L)	4.53 (3.68, 5.48)	4.57 (3.68, 5.89)	0.584
HDL cholesterol (mmol/L)	1.00 (0.82, 1.21)	1.03 (0.81, 1.35)	0.569
LDL cholesterol (mmol/L)	2.72 (2.01, 3.58)	2.73 (1.91, 3.73)	0.877
Albumin (g/L)	39 (33.8, 42.6)	42.7 (35.5, 45.9)	0.028
Triglyceride (mmol/L)	1.51 (0.97, 2.32)	1.93 (1.03, 3.22)	0.111
eGFR (mL/min/1.73 m^2^)	90.095 (37.26,105.749)	85.766 (55.073,101.958)	0.830
RBC (10^12^/L)	4.04 ± 0.91	4.24 ± 0.72	0.228
WBC (10^9^/L)	6.06 (5.2, 7.22)	6.33 (5.41, 7.10)	0.324
PLT (10^9^/L)	212 (178,246)	220 (188,268)	0.417
Hb (g/L)	125 (103.4,139)	130 (112.2,146.2)	0.118
24hTP (g)	1.04 (0.17, 4.58)	1.1 (0.18, 3.52)	0.596
NAG(U/L)	9.9 (5, 15.3)	11.0 (4.9, 20.2)	0.782
URBP (μg/L)	8.75 (5.56, 10.16)	8.16 (4.38, 9.68)	0.024
α1-MG (μg/L)	9.44 (8.01, 10.69)	8.96 (7.7, 10.09)	0.087
β2-MG (μg/L)	6.58 (5.25, 8.27)	6.11 (4.61, 7.13)	0.134^2^

LDL low density lipoprotein, HDL high density lipoprotein, eGFR estimated glomerular filtration rate, RBC red blood cell, WBC white blood cell, PLT platelet, Hb hemoglobin, 24hTP 24 h uric total protein, NAG N-acetyl-beta-D-glucosaminidase, URBP urinary retinol binding protein, α1-MG α1-microglobulin, β2-MG β2-microglobulin.

### Predictors selection

By univariate logistic regression, 14 potential predictors were considered statistically significant among 26 variables for demographic and laboratory test measures. The 14 candidate variables were enrolled to develop the multivariable model. From multivariate logistic regression, three potential factors were identified as significant including eGFR, NAG and URBP (Table 3). The results indicated that diabetic patients with lower eGFR levels, higher α1-MG levels and higher URBP levels were more likely suffering diabetic kidney disease.

**TABLE 3:** Potential risk factors identified by univariate and multivariate logistic regression analysis.

	Univariable multivariable				
	OR (95%CI)	*p*		OR (95%CI)	*p*
Age (years)	1.364 (0.719, 2.586)	0.342			
Female n (%)	1.024 (0.995, 1.054)	0.113			
body mass index (kg/m^2^)	1.012 (0.928, 1.104)	0.785			
Systolic blood pressure (mmHg)	1.063 (1.038, 1.088)	<0.001			
Diastolic blood pressure (mmHg)	1.048 (1.015, 1.081)	0.004			
Serum potassium (mmol/L)	1.332 (0.742, 2.392)	0.337			
Serum sodium (mmol/L)	1.074 (0.987, 1.168)	0.099			
Serum calcium (mmol/L)	0.004 (0.000, 0.046)	<0.001			
Urea nitrogen (mmol/L)	1.554 (1.303, 1.852)	<0.001			
Serum creatinine (mmol/L)	1.058 (1.034, 1.081)	<0.001			
Uric acid (μmol/L)	1.005 (1.002, 1.009)	0.004			
Total cholesterol (mmol/L)	1.058 (0.868, 1.291)	0.577			
HDL cholesterol (mmol/L)	0.511 (0.209, 1.246)	0.140			
LDL cholesterol (mmol/L)	1.068 (0.836, 1.365)	0.599			
Albumin (g/L)	0.836 (0.781, 0.895)	<0.001			
Triglyceride (mmol/L)	1.001 (0.864, 1.159)	0.992			
eGFR (ml/min/1.73 m^2^)	0.924 (0.899, 0.949)	<0.001		0.916 (0.864, 0.972)	0.003
RBC (10^12^/L)	0.306 (0.188, 0.499)	<0.001			
WBC (10^9^/L)	1.081 (0.884, 1.323)	0.449			
PLT (10^9^/L)	1.000 (0.995, 1.005)	0.910			
Hb (g/L)	0.959 (0.943, 0.975)	<0.001			
24hTP (g)	1734113.338 (3873	<0.001			
	658,776307358.8)				
NAG (U/mL)	1.165 (1.096, 1.238)	<0.001			
URBP (mg/mL)	6.327 (3.206, 12.485)	<0.001		6.286 (2.626, 15.047)	<0.001
α1-MG (μg/L)	6.158 (3.652, 10.383)	<0.001		4.622 (1.679, 12.73)	0.003
β2-MG (μg/L)	3.144 (2.210, 4.471)	<0.001			

LDL low density lipoprotein, HDL high density lipoprotein, eGFR estimated glomerular filtration rate, RBC red blood cell, WBC white blood cell, PLT platelet, Hb hemoglobin, 24hTP 24 h uric total protein, NAG N-acetyl-beta-D-glucosaminidase, URBP urinary retinol binding protein, α1-MG α1-microglobulin, β2-MG β2-microglobulin.

### Great diagnostic efficacy

**FIGURE 1:**
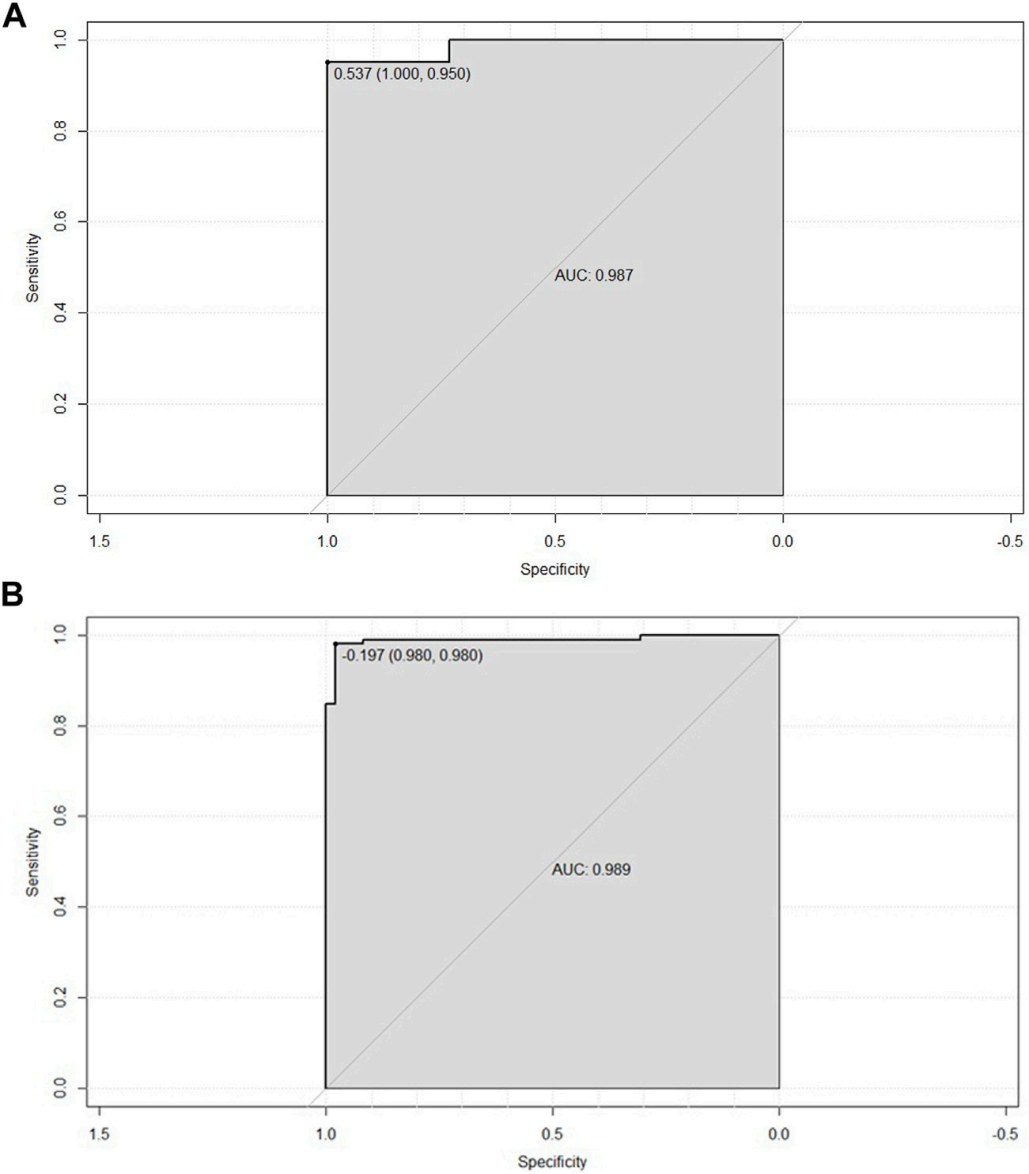
**(A)** ROC curve based on obtained potential risk factors identified by multivariate logistic regression analysis showing area under the curve (AUC) for the nomogram score in the training group. **(B)** ROC curve based on obtained potential risk factors identified by multivariate logistic regression analysis showing area under the curve (AUC) for the nomogram score in the validation group.

**FIGURE 2:**
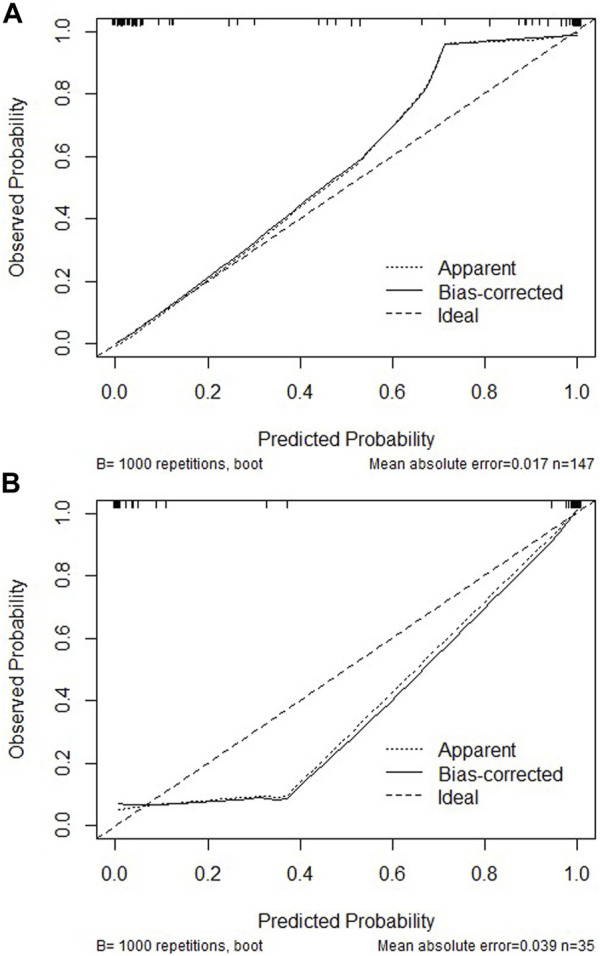
Calibration curves of the DKD incidence risk nomogram prediction in the training cohort **(A)** or validation cohort **(B)**. The *x*-axis represents the predicted DKD incidence risk. The *y*-axis represents the actual diagnosed DKD. The diagonal dotted line represents a perfect prediction by an ideal model. The solid line represents the performance of the nomogram, of which a closer fit to the diagonal dotted line represents a better prediction.

Based on the potential predictors from multivariate logistic regression, we attempt to establish a model for precisely identifying the risk of developing DKD. We drew ROC based on eGFR, α1-MG and URBP to evaluate the diagnostic effectiveness of the model (Figure 1A). The diagnostic model achieved a high accuracy with an AUC of 0.987, which indicates great performance. We subsequently demonstrated its effectiveness in the validation group and the result showed a superior efficiency with an AUC of 0.989 (Figure 1B). For the 182 participants in the array, similar results were observed for calibration curves predicting DKD risk in the training and test groups (Figures 2A, B).

### Construction an individualized nomogram

**FIGURE 3:**
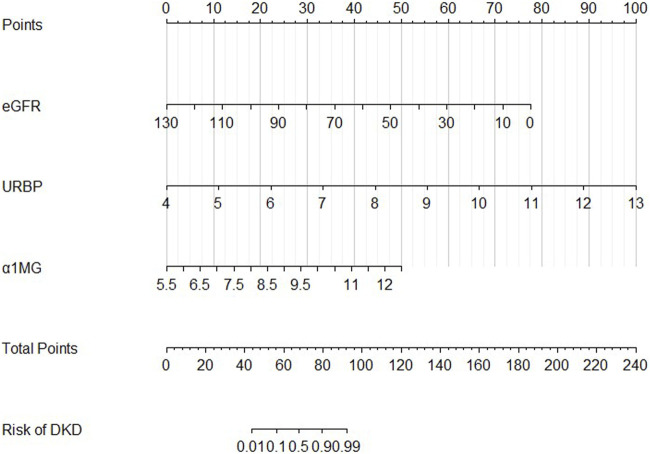
Developed DKD incidence risk nomogram. Values for each variable were expressed as scores by drawing a line up to the “points” line from the corresponding value. Sum the total number of points and mark on the “Total points” line. Draw a straight line to the corresponding “rate” axis to get the possibility of DKD.

**FIGURE 4:**
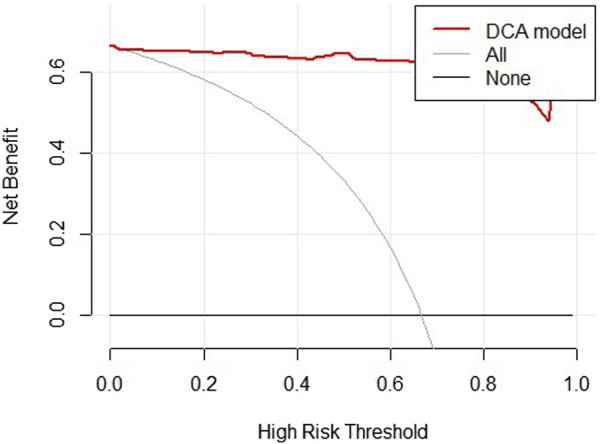
Decision curve analysis for the DKD incidence risk nomogram in training group. The grey line represents the assumption that all patients are diagnosed as DKD. The black solid line represents the assumption that no patients are diagnosed as DKD. The thick red line represents the model.

We constructed the nomogram of our discrimination model based on three obtained predictive variables including eGFR, α1-MG and URBP (Figure 3). The result of decision curve analysis for the nomogram was shown in Figure 4.

### URBP predicted early renal impairment

To investigate whether tubular injury markers could accurately predict the risk of DKD in patients with normal renal function, we screened patients with relatively normal eGFR (≥90 mL/min/1.73 m^2^) in the DKD group and matched eGFR and creatinine in both groups so that there was no statistically significant difference of those two indicators. The basic characteristics between the DM and DKD groups is shown in Table 4. Similarly, we performed univariate and multivariate logistic regression analyses. It suggests URBP (*p* < 0.05) play an important role in early prediction of renal impairment.

**TABLE 4:** Differences in demographic and clinical characteristics between the DM and DKD groups after adjusting eGFR and CREA.

	DM group (N = 25)	DKD group (N = 25)	*p*-value
Age (years)	51.67 ± 11	54.93 ± 10	0.712
Female n (%)	10 (40)	6 (24)	
body mass index (kg/m^2^)	24.88 ± 3.55	26.50 ± 3.48	0.619
Systolic blood pressure (mmHg)	130.08 ± 13.26	138.76 ± 9.13	0.007
Diastolic blood pressure (mmHg)	80.68 ± 8.56	85.76 ± 8.248	0.050
Serum potassium (mmol/L)	4.25 ± 0.38	4.13 ± 0.48	0.648
Serum sodium (mmol/L)	140 (138.5,142.0)	143.0 (139.50,144.0)	0.051
Serum calcium (mmol/L)	2.31 ± 0.13	2.29 ± 0.12	0.676
Urea nitrogen (mmol/L)	5.40 (4.30, 6.80)	5.30 (4.50, 6.80)	0.977
Serum creatinine (mmol/L)	61 (52.5, 73.5)	64 (52.5, 72.5)	0.801
Uric acid (μmol/L)	274 (212.5,327)	298 (247,320)	0.362
Total cholesterol (mmol/L)	4.53 (3.70, 5.06)	3.93 (3.60, 4.76)	0.130
HDL cholesterol (mmol/L)	1.06 (0.80, 1.27)	0.93 (0.73, 1.08)	0.101
LDL cholesterol (mmol/L)	2.77 (1.96, 3.48)	2.48 (1.69, 2.85)	0.123
Albumin (g/L)	42.01 ± 3.17	41.40 ± 4.37	0.432
Triglyceride (mmol/L)	1.51 (0.95, 2.40)	1.68 (1.01, 2.89)	0.594
eGFR (mL/min/1.73 m^2^)	103.69 (95.21,108.99)	102.46 (94.935,107.22)	0.884
RBC (10^12^/L)	4.39 (4.04, 4.76)	4.23 (4.1, 4.81)	0.892
WBC (10^9^/L)	5.7 (5.13, 6.71)	6.4 (5.5, 7.3)	0.162
PLT (10^9^/L)	217 (187,256)	217 (189.5,272.5)	0.720
Hb (g/L)	132.98 ± 13.32	133.29 ± 18.32	0.923
24hTP (g)	0.15 (0.12, 0.31)	0.7 (0.45, 1.88)	<0.001
NAG (U/mL)	7.78 (7.05, 8.50)	9.30 (8.05, 9.79)	<0.001
URBP (mg/mL)	5.56 (5.50, 5.58)	8.75 (7.08, 9.36)	<0.001
α1-MG (mg/L)	2.40 (1.15, 4.93)	10.95 (3.12, 17.76)	<0.001
β2-MG (mg/L)^a^	0.22 (0.15, 0.27)	0.72 (0.30, 1.42)	0.002

^a^ NAG N-acetyl-beta-D-glucosaminidase, URBP urinary retinol binding protein, α1-MG α1-microglobulin, β2-MG β2-microglobulin.

### No relationship with renal progression

In order to study whether the tubular injury markers correlate with kidney prognosis, we collected the follow-up information of eGFR in DKD group. Through correlation analysis, it showed that the level of tubular injury markers was not related to the decline rate of eGFR (Table 5).

**TABLE 5:** Spearman correlation analysis between markers of tubular injury and the degree of glomerular filtration rate decline over a period time.

Tubular injury markers	*p*-value
NAG	0.176
URBP	0.114
α1-MG	0.259
β2-MG	0.344

NAG N-acetyl-beta-D-glucosaminidase, URBP urinary retinol binding protein

## Discussion

In this retrospective case–control study, we collected and analyzed the laboratory examination of 182 patients with DM and DKD. We established a clinical diagnostic model using three indicators including eGFR, and URBP and improved ability to predict risk of DKD. In addition, we found that URBP (*p* < 0.05) may play an important role in early prediction of renal impairment with normal renal function. We also collected follow-up information from part of patients, compared the decrease of eGFR over a period time. And we found the level of tubular injury markers did not correlate with renal prognosis which were rarely studied in related fields.

At present, there are still many shortcomings in the diagnosis of diabetic nephropathy by non-invasive means alone in clinical work. Recent studies have shown that some diabetic patients with normal proteinuria have progressive renal dysfunction, known as normoalbuminuric diabetic kidney disease (NADKD) (Chen et al., 2017). Therefore, it is very important to find biomarkers that can identify diabetic nephropathy in the early non-invasive stage and accurately.

This study found that NAG was the risk factor for DKD in patients with T2DM. N-acetyl-b-D-glucosaminidase (NAG) is a lysosomal brush border enzyme that is localized in the microvilli of renal tubular epithelial cells. Because NAG has a large molecular weight (>130 kD), it does not filter through the glomeruli unless tubular injury occurs (Liangos et al., 2007; Moresco et al., 2013). Urine NAG level were significantly higher in DKD patients than those in NDKD (Non-diabetic kidney disease) patients in our study (*p* < 0.001). Besides, our study found that NAG did not correlate greatly with the rate of glomerular decline. Furthermore, a similar study from Japanese showed that in a cohort of patients with DKD confirmed by renal biopsy, assessment of urinary NAG levels did not add prognostic value (Mise et al., 2016b).

RBP is synthesized and secreted by the liver to transport retinol from hepatocytes to surrounding tissues. It carries vitamin A into cells, releases it and then becomes denatured and inactivated. This free binding protein can be filtered in the glomerulus, most of which is reabsorbed by the proximal tubular epithelial cells and broken down for tissue use, and only a small amount is excreted in the urine (Abbasi et al., 2020a). It is a well-recognized biomarker of proximal tubular dysfunction (PTD) in diseases characterized by the renal Fanconi syndrome such as those associated with plasma cell dyscrasias (Vignon et al., 2017). Fatemeh Abbasi’s research results showed that URBP was related to the severity of the disease, and the URBP level of patients with large albuminuria was significantly higher than that of patients with moderate albuminuria, and it was still significant after adjusting for other metabolic factors (all *p* < 0.01) (Abbasi et al., 2020b), which is similar to what we found in our study. URBP levels play an essential role in our model whether renal function is normal or not.

α1-MG is a low-molecular-weight glycoprotein, which is a hydrophobic ligand-binding protein. Like RBP, the free form is easily filtered by the glomerulus, but almost all of it is absorbed and metabolized by the renal tubules. Thus, increased urinary excretion of α1-MG suggests impaired proximal renal tubule function (Hong et al., 2003). There is a study indicate urinary α1-MG was also increased before the onset of microalbuminuria but they failed to identify α1-MG as early diagnostic markers for DKD (Zhang et al., 2019), however, in our study, we found that urinary α1-MG can be used as an early predictor of the risk of DKD, but we failed to find a role for urinary α1-MG in the prognosis of DKD. The relationship between α1-MG and development of DKD requires further high-quality, multicenter, prospective studies.

β2-MG is a 99 amino acid single chain polypeptide with a molecular mass of 11,800. Its filtration in the glomeruli and reabsorption in the renal tubules are like to RBP that can be filtered freely from glomeruli and almost completely absorbed by renal tubules (Qin et al., 2019). Xu Jiang’ study indicates that the excretion of β2-MG increases in the early course of DKD, whereas urinary albumin excretion is normal in patients with diabetes. Also, β2-MG levels were elevated in diabetic patients with relatively normal renal function (eGFR ≥90 mL/min/1.73 m^2^) compared with nondiabetic control subjects. These data illustrate that tubular markers have an important role in the early development of renal function impairment in diabetic patients (Levey et al., 2011). In other studies, we found that urinary β2-MG levels were also significantly higher in DKD patients than in DM patients (Papale et al., 2010). Although β2-MG is not as sensitive to diagnosis as other markers, previous studies have shown a strong negative correlation between β2-MG and glomerular filtration rate. It may be a sensitive marker for predicting progression or prognosis of kidney function injury (Foster et al., 2015; Chen et al., 2018; Colombo et al., 2019). But in our study, there was no correlation between β2-MG and the rate of decline of glomerular filtration rate, and there was no effect in predicting kidney outcome either.

The decision curve showed the clinical utility of our model, indicating it may be beneficial for clinicians to diagnose the disease by using our model. The results of decision curves suggest the good clinical application value of our model.

In summary, our model can improve the ability to predict the risk of DKD in clinical work. Through the estimation of individual risks, clinicians and patients can take more precise measurements in lifestyle monitoring and medical intervention. Compared to renal biopsy, our nomogram is noninvasive and does not have any contraindications. In addition, laboratory parameters in nomograms are easily measured. There are also several limitations in our current study. First, our sample size is not large. Second, it is a single-centered study. Third, the diagnosis of diabetic nephropathy is based on clinical criteria and is not as precise as renal biopsy.

## Data Availability

The raw data supporting the conclusion of this article will be made available by the authors, without undue reservation.
